# Clinical efficacy of Enhanced Recovery After Surgery in intervening patients with ureteral calculi complicated with infection and its impact on quality of life

**DOI:** 10.12669/pjms.41.10.11946

**Published:** 2025-10

**Authors:** Dan Shen, Xinyao Li, Shuang Pan, Hongmei Li, Jingyan Wang

**Affiliations:** 1 Dan Shen Department of Urology, Affiliated Hospital of Hebei University, Baoding 071000, Hebei, China; 2 Xinyao Li Hebei University School of Basic Medical Sciences, Baoding 071000, Hebei, China; 3 Shuang Pan Department of Urology, Affiliated Hospital of Hebei University, Baoding 071000, Hebei, China; 4 Hongmei Li Department of Emergency Medicine, Affiliated Hospital of Hebei University, Baoding 071000, Hebei, China; 5 Jingyan Wang Department of Urology, Affiliated Hospital of Hebei University, Baoding 071000, Hebei, China

**Keywords:** Enhanced recovery after surgery, Ureteral calculi complicated with infection, Percutaneous nephrolithotripsy, Quality of life, Clinical efficacy

## Abstract

**Objective:**

To evaluate the clinical efficacy of ERAS (enhanced recovery after surgery) in intervening patients with ureteral calculi complicated with infection and its impact on quality of life.

**Methodology:**

This prospective study included 120 ureteral calculi patients with infection admitted to Affiliated Hospital of Hebei University from September 2023 to December 2024. They were randomly divided into control and experimental groups (60 cases each). The control group received standard perioperative care, while the experimental group received ERAS in addition. The groups were compared for emotional status, clinical outcomes (calculus clearance, complications like hemorrhage, infection and abdominal effusion), recovery, quality of life and satisfaction.

**Results:**

The experimental group showed significantly lower the Self-rating Anxiety Scale (SAS) and the Self-rating Depression Scale (SDS) scores than the control group post-intervention (P=0.00). Their calculus clearance rate was higher (P=0.03), while complication rates were lower (P=0.02). Postoperative recovery times (nephrostomy tube retention, diet recovery, first exsufflation, ambulation and hospitalization) were significantly shorter (P=0.00). The experimental group also had better KPS and SF-36 scores (P=0.00) and higher satisfaction (100% vs. 88%, P=0.00).

**Conclusion:**

ERAS is a safe, effective approach that reduces negative emotions and complications, improves calculus clearance, enhances quality of life, boosts satisfaction and accelerates recovery.

## INTRODUCTION

Ureteral calculi are a common disease of the urinary system in which the incidence accounts for 40%-50%. The incidence of the disease has been witnessed in recent years to be on the rise year by year with the changes in lifestyle and dietary structure.^1^ Ureteral calculi are pathogenetically complicated and result from diet, lifestyle habits, metabolic capacity of the body and environmental factors. In the case of a calculus diameter greater than 2.5cm, it often leads to severe hydronephrosis and infection due to complete obstruction on the affected side.^2^

For infected large ureteral calculi, preferred surgical options include PCNL (Percutaneous nephrolithotripsy), laparoscopic lithotomy and open surgery. Although PCNL is minimally invasive with benefits like reduced bleeding and faster recovery, it induces significant pain and impairs recovery due to surgical trauma and anesthesia.^3^ ERAS refers to a series of measures taken to minimize the occurrence of surgical stress and postoperative complications, reduce the morbidity and mortality rate, speed up patients’ postoperative recovery and shorten hospital stays with a variety of effective methods in the preoperative, intraoperative and postoperative periods.^4^

ERAS nursing is an innovative perioperative approach that employs targeted clinical interventions to reduce stress responses, complications and pain while accelerating recovery. As a core component of enhanced recovery protocols, it embodies patient-centered care principles. With ERAS’s evolving surgical applications, nursing practices require corresponding updates.^5^

## METHODOLOGY

This prospective study enrolled 120 infected ureteral calculus patients at Hebei University Affiliated Hospital (September 2023 to December 2024), randomly allocated equally to control and experimental groups. Demographic data showed comparable distributions between groups (experimental: 35M/25F, 58.35±6.74 years; control: 33M/28F, 57.59±6.53 years) without significant differences (P>0.05, Table-I). Both groups underwent standardized PCNL (with preoperative DJ stent placement for one month and confirmed urine WBC <100/HP) performed by a single surgeon. Controls received conventional perioperative care: (1) preoperative health education/psychological counseling; (2) postoperative monitoring (fever, pain, abdominal distension, nausea/vomiting) with symptomatic management.

**Table-I:** Comparative analysis of the general data of the experimental group and the control group (*χ̅*±*S*) n=60.

Indicators	Experimental group	Control group	t/χ^2^	P
Age (yrs.)	58.35±6.74	57.59±6.33	0.63	0.52
Male (cases, %)	35 (58%)	33 (55%)	0.14	0.71
Calculus location (left, %)	31 (52%)	29 (48%)	0.13	0.72
Calculus diameter (cm)	2.85±0.16	2.81±0.11	1.60	0.11
Degree of infection (moderate, %)	53 (88%)	55 (92%)	0.37	0.54

P>0.05.

### Ethical Approval:

The study was approved by the Institutional Ethics Committee of Affiliated Hospital of Hebei University (No.: HDFYLL-KY-2023-161; Dated: September 25, 2023) and written informed consent was obtained from all participants.

### Inclusion criteria:

- Patients diagnosed with ureteral calculi by imaging examination (ultrasound, X-ray, CT, etc.).
- Those with moderate or severe infection requiring preoperative indwelling of DJ tube for drainage.^6^
- Those aged 45~75 years.
- Those with calculus diameter >2.5 cm who required percutaneous nephrolithotomy.^7^
- Those with clinical symptoms such as fever, lumbago, soreness of the lower back, nausea and vomiting.
- Those with normal cognitive ability who could cooperate with the study.
- Those with complete clinical data.
- Those who signed an informed consent form.

### Exclusion criteria:

- Patients with calculus volume <2.5 cm and suitable for ureteroscopic or flexible ureteroscopic surgery.
- Those combined with congenital malformations that require surgical correction.
- Those with combined heart, brain, liver, kidney and other important organ diseases that cannot be satisfactorily corrected.
- Those with coagulation disorders.
- Those who were unable to complete the study due to the presence of psychiatric disorders or cognitive or behavioral abnormalities.
- Those who withdrew from the study midway.

### The experimental group received additional ERAS interventions:

- ***Psychological care:*** Preoperative anxiety/depression assessment and tailored counseling were provided, along with detailed surgical education to improve patient understanding.
- ***Prone positioning training:*** Patients underwent progressive training (30min→3hr) with abdominal padding to simulate PCNL positioning, mitigating respiratory/cardiovascular risks.
- ***Preoperative optimization:*** Adhering to ERAS principles, patients received liquid diet until the night before surgery and 10% glucose-saline solution 4hr preoperatively to reduce fasting duration.

### Postoperative ERAS Protocol:

- ***Early Oral Intake:*** Initiated with 50-100 mL warm water at two hours post-op, progressed to semi-liquid diet at eight hours (if bowel sounds present, no distension) and regular diet at 24 hours.
- Mobilization.
- 0-6h: Head-elevated position with assisted limb exercises.
*Post-op Day 2: * Semi-recumbent positioning with guided breathing/coughing exercises and muscle training.
- Multimodal analgesia enabled early ambulation.

### Catheter Management:

- Early removal based on drainage/recovery status.
- Routine tube patency checks (color/volume), clot prevention via milking and scheduled bag replacement.

### Health Guidance:

- Fluid intake >2500 mL/day (hematuria patients prioritized).
- High-fiber diet, reduced animal protein/salt.
- Double-J tube care education.

### Observation Indicators:

- Psychological status: Assessed using SAS and SDS (lower scores indicate better status).^8^
- Surgical outcomes: Calculated stone clearance rate and complication rates (hemorrhage, infection, abdominal effusion).
- Recovery parameters: Recorded nephrostomy tube duration, dietary recovery, first flatus, ambulation and hospitalization time.
- Quality of life: Evaluated by KPS and SF-36 scales.^9^
- Patient satisfaction: Measured using PSQ-18 (total).^10^
- Satisfaction=[very+relatively+satisfied]/total×100%).

### Statistical analysis:

All analyses were performed using SPSS 20.0 (IBM Corp., Chicago, IL, USA). Continuous variables (quantitative data) were presented as mean ± standard deviation (± SD) and analyzed using: Independent samples *t*-test (between-group comparisons) and Paired *t*-test (within-group comparisons). Categorical variables were compared using the χ² test. A two-tailed P < 0.05 defined statistical significance.

## RESULTS

No statistically significant difference was found between the levels of SAS and SDS of the experimental group and the control group before the intervention (*P*>0.05). After the intervention, the levels of SAS and SDS in the experimental group were obviously lower than those in the control group after the intervention, with statistically significant differences (*P*=0.00) (Table-II).

**Table-II:** Comparative analysis of the emotional state of the two groups before and after the intervention (*χ̅*±*S*) n=60.

Indicators		Experimental group*	Control group	t	p
SAS	Before intervention	64.06±7.42	63.89±7.53	0.13	0.90
	After intervention*	41.25±7.30	62.85±7.85	16.43	0.00
SDS	Before intervention	65.35±7.06	66.02±7.33	0.51	0.60
	After intervention*	43.26±7.71	62.08±7.68	13.56	0.00

* P<0.05

The calculus clearance rate of the experimental group was noticeably higher than that of the control group, with a statistically significant difference (*P*=0.03), while the incidence of surgical complications in the experimental group was significantly lower than that of the control group, with a statistically significant difference (*P*=0.02) (Table-III).

**Table-III:** Comparative analysis of surgical outcomes between the two groups (*χ̅*±*S*) n=60.

Group	Calculus clearance rate (%)	Incidence of surgical complications (%)			
		Hemorrhage	Infection	Abdominal effusion	Incidence rate
Experimental group	97% (58/60)	0	2	0	2 (20%)
Control group	85% (51/60)	3	4	2	9 (42%)
*χ* ^2^	4.90				4.23
*P*	0.03				0.02

P<0.05.

The postoperative nephrostomy tube retention time, diet recovery time, first exsufflation time, ambulation time and postoperative hospitalization time of the experimental group were significantly shorter than those of the control group, with statistically significant differences (*P*=0.00) (Table-IV).

**Table-IV:** Comparative analysis of postoperative recovery of the two groups (*χ̅*±*S*) n=60.

Group	Nephrostomy tube retention time (h)	Diet recovery time (h)	First exsufflation time (h)	Ambulation time (h)	Postoperative hospitalization time (h)
Experimental group	22.57±4.02	22.09±8.90	15.76±3.08	19.64±5.06	57.62±4.79
Control group	31.84±4.79	45.76±10.33	24.63±3.27	25.87±6.03	75.17±8.54
*t*	10.89	13.24	16.43	5.95	14.78
*P*	0.00	0.00	0.00	0.00	0.00

P<0.05.

No statistically significant difference was found between the KPS score and SF-36 quality of life score of the two groups before the intervention (*P*>0.05). After the intervention, the above indicators in the experimental group improved significantly compared to the control group, with statistically significant differences (*P*=0.00) (Table-V).

**Table-V:** Comparative analysis of quality of life scores between the two groups before and after treatment (*χ̅*±*S*) n=60.

Indicators		Experimental group	Control group	t	p
KPS score	Before intervention	64.72±9.38	64.69±9.16	0.32	0.75
	After intervention*	73.59±10.31	67.30±10.18	3.36	0.00
SF-36 score	Before intervention	45.63±7.33	46.02±7.14	0.30	0.77
	After intervention*	62.04±8.46	53.37±7.79	5.84	0.00

* P<0.05.

Patient satisfaction in the experimental group was 100%, which was significantly higher than 88% in the control group, with a statistically significant difference (P=0.00) (Table-VI).

**Table-VI:** Comparative analysis of patient satisfaction between the two groups (*χ̅*±*S*) n=60.

Group	Very satisfied	Relatively satisfied	Satisfied	Uncertain	Unsatisfied	Total satisfaction*
Experimental group	40	8	12	0	0	60 (100%)
Control group	23	17	13	4	3	53 (88%)
*χ* ^2^						7.43
*P*						0.00

* P<0.05.

## DISCUSSION

Enhanced Recovery After Surgery (ERAS), a multimodal perioperative care protocol first introduced by Danish surgeon Kehlet H,^11^ focuses on optimizing patient outcomes through evidence-based interventions. This approach effectively minimizes surgical stress responses, reduces complication rates, shortens hospital stays and enhances patient recovery while improving overall satisfaction with care.^12^ The ERAS protocol spans the entire perioperative period, promoting better coordination between medical and nursing care while standardizing clinical practices.^13^-^15^

Ding et al.^16^ demonstrated the clinical feasibility and superiority of the ERAS nursing model in urological, hepatobiliary and biliary surgeries. In our study, we implemented the ERAS protocol for patients undergoing percutaneous nephrolithotripsy for ureteral calculi complicated by infection. The intervention included preoperative psychological counseling, positional training and optimized preparation, along with postoperative early feeding, ambulation, catheter care and comprehensive health education, yielding significant clinical benefits.

### The ERAS nursing model offers multiple advantages for complex renal calculi management:

- Preoperative patient education and targeted psychological intervention reduce preoperative anxiety and improve satisfaction;
- Gradual positional training enhances patient tolerance to surgical positioning, minimizing intraoperative discomfort and improving surgical outcomes;
- Patient-specific preoperative preparation and reduced fasting duration improve surgical tolerance;
- Early postoperative oral intake and mobilization promote gastrointestinal recovery and shorten hospitalization;
- Meticulous catheter care prevents dislodgement and infection;
- Enhanced health education, including fluid intake and dietary modifications, facilitates calculus clearance and recovery.

Our results demonstrate significantly lower SAS and SDS scores in the ERAS group compared to controls (P=0.00), with 100% patient satisfaction versus 88% in controls (P=0.00). These findings suggest that preoperative psychological intervention effectively alleviates anxiety and depression while improving nursing satisfaction.^17^

The ERAS group showed significantly shorter durations in nephrostomy tube retention, dietary recovery, first flatus, ambulation and postoperative hospitalization (P=0.00). This indicates that early feeding and mobilization accelerate recovery of gastrointestinal function and reduce drainage tube dependence. The calculus clearance rate was significantly higher in the ERAS group (97% vs 85%, P=0.03), with fewer surgical complications (20% vs 42%). Positional training improved patient adaptability to prone positioning, reducing intraoperative complications. Grosso et al.^18^ similarly reported that preoperative positional training reduces intraoperative stress and improves surgical outcomes. Our data also revealed significant improvements in KPS and SF-36 quality of life scores in the ERAS group (P=0.00), underscoring the model’s role in enhancing postoperative recovery.

These benefits likely stem from the comprehensive, multidisciplinary nature of ERAS care and reinforced health education, which collectively reduce complications and improve quality of life - consistent with findings by Do-Wyeld et al.^19^ Additionally, Wang et al.^20^ demonstrated that ERAS mitigates psychological stress and inflammatory responses, thereby reducing systemic stress and postoperative pain.

### Limitations:

The findings of this study have to be seen in light of some limitations: the sample size is small and only covers patients who underwent percutaneous nephrolithotripsy for ureteral calculi. To address this, the sample size will be further expanded, the follow-up period will be extended and other treatment options such as ureteroscopic lithotripsy and ureteral flexible lithotripsy will be included in future clinical work. With this endeavor, we aim to further enrich the study by objectively evaluating the significance of ERAS in different surgeries for patients with calculi, so as to benefit a larger number of patients.

## CONCLUSIONS

ERAS nursing boasts various benefits for patients with ureteral calculi complicated with infection who underwent percutaneous nephrolithotomy, such as eliminating the adverse emotions of patients in the perioperative period, facilitating postoperative recovery, improving the calculus clearance rate, decreasing the incidence of postoperative complications and improving the quality of life of the patients, thus increasing patients’ satisfaction with the nursing care, which is of certain clinical value.

### Authors’ Contributions:

**DS** and **XL**: Literature search, Conceived, designed the study and Review.

**SP, HL and JW:** Collected the data, performed the analysis, critical review, were involved in the writing of the manuscript.

All authors have read and approved the final manuscript and are responsible for the integrity of the study.
